# CARE’S ECHO: THE IMPACT OF CAREGIVING DURING COVID-19 ON OLDER WORKERS’ FINANCIAL, HEALTH, AND JOB OUTCOMES

**DOI:** 10.1093/geroni/igae098.1103

**Published:** 2024-12-31

**Authors:** Christina Matz, Patrick Lai, Barbara Mendez Campos

**Affiliations:** Boston College, Chestnut Hill, Massachusetts, United States; Boston College, Chestnut Hill, Massachusetts, United States; Boston College, Chestnut Hill, Massachusetts, United States

## Abstract

Informal caregivers are a critical resource to their loved ones and an essential component of the health care system in the United States. In the context of an ongoing care crisis, it is more important than ever to understand the effect of informal care responsibilities on individuals and their families as they age to develop appropriate solutions. This study examines how caregiving responsibilities during COVID-19 affected older workers’ financial well-being, health, job turnover, and employment experiences. Using 2020 Health and Retirement Study (HRS) data and the 2021 HRS Perspectives on the Pandemic survey, we examined older workers aged 50 and above. Our logistic regression analysis focused on two caregiving scenarios: 1) individuals with a spouse or partner with ADL/IADL limitations, and 2) those who paused work for at least two weeks due to caregiving during the pandemic. Our findings reveal significant challenges for caregivers. Spousal caregivers were 1.65 times more likely to face financial difficulties and 1.87 times more likely to have health issues due to delayed care compared to non-caregivers. Those who had to stop working for 2 weeks or more for caregiving reported even higher risks (2.69 times for financial difficulty and 3.29 times for health issues). Caregivers also experienced greater job stress, safety concerns, and decreased job satisfaction. The pandemic has illuminated the struggles of older working caregivers, emphasizing the need for supportive workplace policies and societal interventions. These measures are essential to support caregivers, who often sacrifice their well-being and career progress for their caregiving roles.

